# Relationship between plasma homocysteine and chronic kidney disease in US patients with type 2 diabetes mellitus: a cross-sectional study

**DOI:** 10.1186/s12882-022-03045-6

**Published:** 2022-12-31

**Authors:** Zilong Shen, Zhengmei Zhang, Wenjing Zhao

**Affiliations:** 1 Department of Nephrology, Beijing Hospital of Traditional Chinese Medicine, Capital Medical University, 23 Art Museum Back Street, Dongcheng District, Beijing, 100010 China; 2 Department of TCM Internal Medicine, Huguosi TCM Hospital, Affiliated with Beijing University of Chinese Medicine, Beijing, 100700 China

**Keywords:** Homocysteine, Chronic kidney disease, Type 2 diabetes, Kidney disease progression

## Abstract

**Aims:**

This cross-sectional study aimed to investigate the association between plasma homocysteine (Hcy) and chronic kidney disease (CKD) in US patients with type 2 diabetes mellitus (T2DM).

**Methods:**

We used data from the 2003–2006 National Health and Nutritional Examination Surveys (NHANES). CKD was defined as an estimated glomerular filtration rate < 60 ml/min/1.73 m^2^ and/or urinary albumin-creatine ratio ≥ 3 mg/mmol.

**Results:**

This study included 1018 patients with T2DM. The mean Hcy value was 10.2 ± 4.6 μmol/L. Among the patients, 417 (40.96%) had hyperhomocysteinemia (HHcy) and 480 (47.15%) had CKD. The Hcy level was higher in patients with CKD than in those without CKD. Compared to patients with normal Hcy, those with HHcy were older and had worse renal function. After full multivariate adjustment, HHcy was positively associated with the risk of CKD in US patients with T2DM (OR, 1.17; 95% CI, 1.11–1.22; *P* <  0.001), which for women was 1.15 (95% CI, 1.08 ~ 1.23; *P* <  0.001) and for men was 1.18 (95% CI, 1.1 ~ 1.27; *P* <  0.001).

**Conclusions:**

HHcy was independently associated with CKD in patients with T2DM. Further prospective studies are warranted to investigate the effect of Hcy on CKD in patients with T2DM.

## Introduction

Type 2 diabetes mellitus (T2DM) is the most common type of diabetes and accounts for over 90% of all diabetes cases worldwide. An estimated 537 million adults aged 20–79 years worldwide (10.5% of all adults in this age group) have diabetes according to the latest International Diabetes Federation (IDF) Diabetes Atlas [1]. Up to 40% of patients with diabetes have comorbidity of chronic kidney disease (CKD), which has the risk of progression to end-stage renal disease (ESKD) and cause high morbidity, mortality, and poor quality of life [2]. The patients with ESKD must receive maintenance dialysis or kidney transplantation to survive, leading to a heavy burden on the health system globally [3]. In addition, CKD patients with diabetes would be at a higher risk of cardiovascular (CV) disease, including myocardial infarction, ischaemic stroke, and all-cause mortality than kidney disease or diabetes itself.

Homocysteine (Hcy) may play a role in the development of cardiovascular (CV) diseases. The role of Hcy in the development of the vascular complications associated with DM was not clearly defined [4]. The 2006 US Stroke guidelines clearly indicated that plasma Hcy > 10 μmol/L was defined as hyperhomocysteinemia (HHcy) [5]. HHcy could reflect the abnormal state of body metabolism for the sake of its damaging effect on cells, tissues and organs [6]. It was an important risk factor for the occurrence of many chronic diseases and regarded as one of the important indicators for many physical illnesses, such as hypertension, hyperlipidemia and hyperglycaemia [7]. Hcy appeared to cause endothelial cell damage and influence the activity and production of coagulation factors [8]. Furthermore, several studies have found that Hcy was associated with eGFR and CKD in the general population [9]. However, few studies have reported the association between Hcy and CKD in patients with diabetes.

The aim of this study was to assess the association between Hcy and CKD in T2DM patients. The various confounding factors which had the possibility to affect the progression to CKD would be adjusted in the cross-sectional study.

## Materials and methods

### Data source and study population

**Fig. 1:**
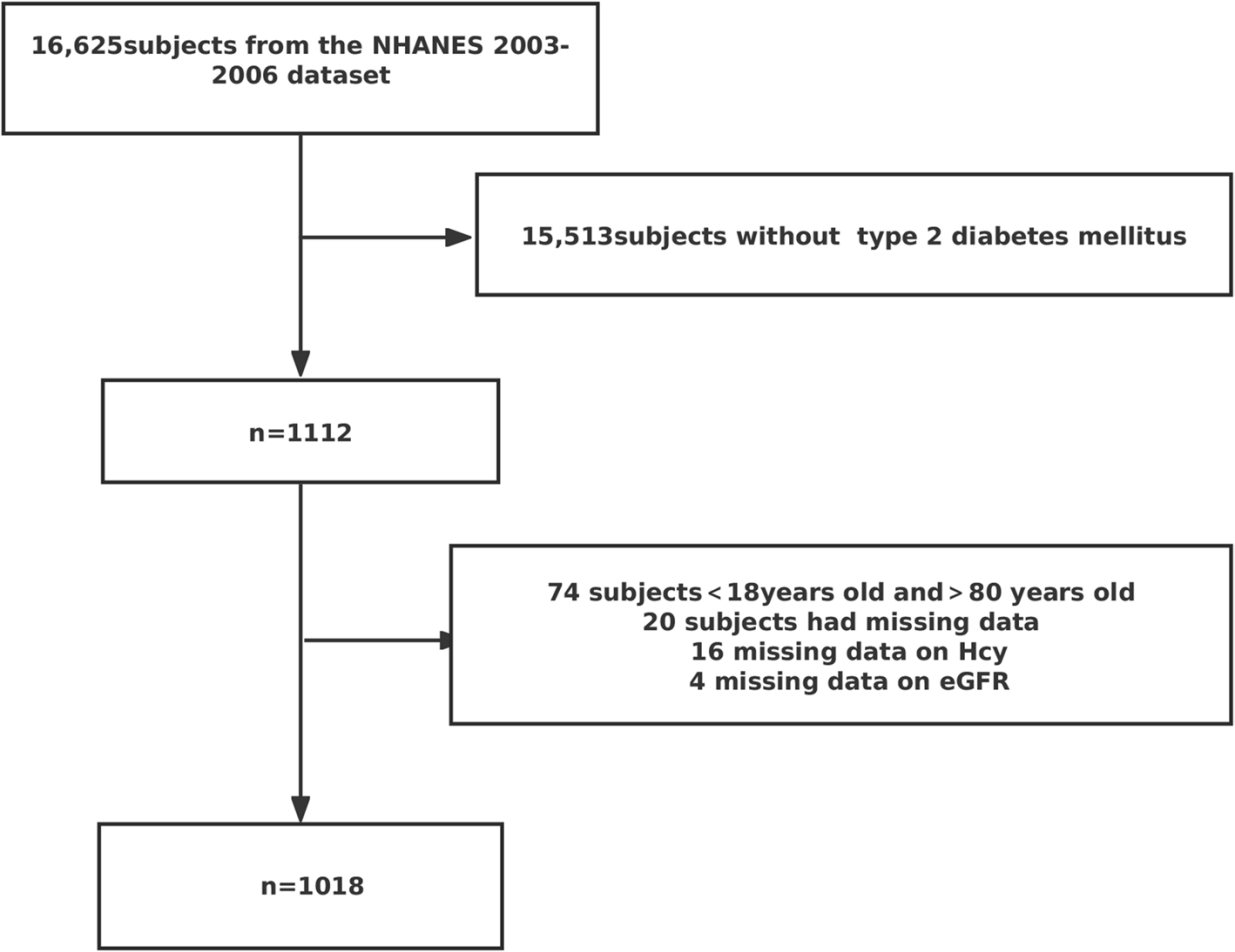
Flowchart of the 1018 subjects included from 16,625 subjects in the NHANES 2003–2006 dataset. Abbreviation: Hcy homocysteine; eGFR, estimated glomerular filtration rate; NHANES, National Health and Nutrition Examination Survey

A NHANES database was used for this study. NHANES is a nationwide population-based survey of the US that has been conducted to assess the health and nutritional status of the US population since 1999. In our research, NHANES data from 2003 to 2004 and 2005–2006 have been chosen because of the integrity of these two time periods for Hcy measurements. Of a total of 16,625 patients, 1112 type 2 diabetes patients were included in this study. A total of 74 patients younger than 18 and older than 80 were excluded. Twenty patients were excluded because 16 had missing data on Hcy and 4 had missing data on eGFR. Finally, the data from 1018 participants were employed for this study (Fig. 1). The research method was cross-sectional study. NHANES was approved by the US National Center for Health Statistics Research Ethics Review Board, and all participants provided informed consent. The Strengthening the Reporting of Observational Studies in Epidemiology (STROBE) statement were followed when reporting this cross-sectional study [10].

### Data collection

We evaluated both clinical characteristics (sex, age, and race) and biochemical parameters (levels of triglycerides [TG], cholesterol [CHO], albumin [ALB], blood urea nitrogen [BUN], serum creatinine [Scr], uric acid [UA], HbA1c, urinary albumin-creatinine ratio [ACR] and plasma homocysteine [Hcy]) collected from the NHANES Laboratory Data. The TG, CHO, ALB, BUN, Scr, and UA analyses were performed using a Beckman Synchron LX20 (Beckman Coulter, Inc).HbA1c measurements were performed by the Diabetes Laboratory at the University of Minnesota using a Tosoh A1c 2.2 Plus Glycohemoglobin Analyser (Tosoh Medics, Inc., San Francisco, CA). Hcy in plasma was measured by the Abbott Homocysteine (Hcy) assay, which is a fully automated fluorescence polarization immunoassay (FPIA) from Abbott Diagnostics.

The albumin in the urine was measured via a fluorescent immunoassay. The urinary creatinine analysis uses a Jaffé rate reaction, in which creatinine reacts with picrate in an alkaline solution to form a red creatinine-picrate complex.

### Measurement of chronic kidney disease

We used ACR and eGFR values as parameters to determine CKD [11]. To calculate the eGFR, the following CKD-EPI formula was applied [12]:

eGFR _CKD-EPI_ (mL/min/1.73 m^2^).

GFR = a × (Scr/b) c × (0.993)^age^.

In this formula, Scr denotes the serum creatinine level (μmol/L);a is 166 for black females and 163 for males;a is 144 for non-black females and 141 for males; b is 0.7 for females and 0.9 for males, and c is − 0.329 for females with serum creatinine ≤62 μmol/L and − 0.411 for males with serum creatinine ≤80 μmol/L. c is − 1.209 for females with serum creatinine≥62 μmol/L and for males with serum creatinine ≥80 μmol/L. The ACR was calculated as the urinary albumin/creatinine ratio. Participants with an ACR above 3 mg/mmoL or an ALFF below 60 mL/min/1.73 m^2^ were classified as CKD patients according to the Kidney Disease Outcome Quality Initiative clinical practice guidelines for CKD.

### Statistical analysis

Data were presented as the mean ± standard deviation (SD) for normally distributed variables or the median (interquartile range) for nonnormally distributed variables and as the frequency or percentage for categorical variables. For the baseline characteristics analysis, the significant differences between two groups were tested based on t test, nonparametric tests for continuous variables and chi-square or Fisher tests for categorical variables. Multiple logistic regression analysis was performed to evaluate the associations between Hcy and CKD, with the results expressed as odds ratios (ORs) and 95% confidence intervals (CIs). The covariates entered into the model were based on univariate analyses and a literature review [13]. All analyses were performed using R Statistical Software (http://www.R-project.org, The R Foundation) and the Free Statistics analysis platform. *P* values< 0.05 (two-tailed) were considered statistically significant.

## Results

### Clinical and biochemical characteristics of the study subjects

The baseline clinical characteristics of the included patients were summarized in Table 1. The mean age of the included patients was 59.9 ± 13.1 years, and 51.2% were male. Of all patients, 480 (47.1%) had CKD. Patients with CKD were older (*P* <  0.001). Levels of Hcy (*P* <  0.001), ACR (*P* <  0.001), Scr (*P* <  0.001), BUN (*P* <  0.001) and UA (*P* <  0.001) were higher in patients with CKD than in those without CKD. No significant differences were found in the proportion of race, HbA1c, TG and CHO between the two groups. Table 2 compared the characteristics of the normal plasma homocysteine and hyperhomocysteinemia groups in which the plasma homocysteine concentration exceeded 10 μmol/L. Compared with patients with normal Hcy, those with HHcy were older (*P* <  0.001). They also had higher levels of ACR and worse renal function (*P* <  0.001) but had lower HbA1c (*P* <  0.001) and CHO (*P* = 0.002).

**Table 1:** Characteristics of the study participants grouped by CKD status

Variables	Total (*n* = 1018)	0 (*n* = 538)	1 (*n* = 480)	p
Male, n (%)	521 (51.2)	249 (46.3)	272 (56.7)	0.001
Age, years	59.9 ± 13.1	57.0 ± 13.3	63.1 ± 12.0	< 0.001
Race, n (%)				0.114
Mexican American	290 (28.5)	162 (30.1)	128 (26.7)	
Other Hispanic	30 (2.9)	18 (3.3)	12 (2.5)	
Non-Hispanic White	378 (37.1)	207 (38.5)	171 (35.6)	
Non-Hispanic Black	278 (27.3)	128 (23.8)	150 (31.2)	
Other Race	42 (4.1)	23 (4.3)	19 (4)	
HbA1c, %	7.5 ± 1.8	7.4 ± 1.7	7.6 ± 1.9	0.091
ALB, g/L	40.7 ± 3.7	41.2 ± 3.2	40.2 ± 4.1	< 0.001
BUN, mmol/L	5.6 ± 3.2	4.6 ± 1.5	6.9 ± 4.0	< 0.001
Scr, μmol/L	91.3 ± 55.1	73.7 ± 15.5	111.0 ± 73.7	< 0.001
eGFR, ml/min/1.73 m^2^	82.4 ± 39.7	94.4 ± 35.2	69.1 ± 40.1	< 0.001
ACR, mg/mmol	1.6 (0.8, 5.1)	0.9 (0.6, 1.5)	5.4 (2.7, 15.6)	< 0.001
CHO, mmol/L	5.2 ± 1.3	5.2 ± 1.2	5.1 ± 1.4	0.305
TG, mmol/L	2.2 ± 2.1	2.1 ± 1.9	2.3 ± 2.2	0.068
UA, μmol/L	334.2 ± 94.3	310.0 ± 79.5	361.2 ± 101.9	< 0.001
Hcy, μmol/L	10.2 ± 4.6	8.8 ± 3.0	11.8 ± 5.5	< 0.001

Abbreviations: *CKD* Chronic kidney disease, *ALB* Albumin, *BUN* Blood urea nitrogen, *Scr* Serum creatinine, *ACR* Albumin-creatinine ratio, *CHO* Cholesterol, *TG* Triglycerides, *UA* Uric acid, *Hcy* Plasma homocysteine concentrations

Data are shown as the mean ± standard deviation, number (percentage) or median (interquartile range)

**Table 2:** Characteristics of the study participants grouped by with and without hyperhomocysteinemia

Variables	Total (*n* = 1018)	Normal Hcy (*n* = 601)	HHcy (*n* = 417)	p
Male, n (%)	521 (51.2)	271 (45.1)	250 (60)	< 0.001
Age,	59.9 ± 13.1	56.0 ± 13.5	65.5 ± 10.0	< 0.001
Race, n (%)				< 0.001
Mexican American	290 (28.5)	194 (32.3)	96 (23)	
Other Hispanic	30 (2.9)	27 (4.5)	3 (0.7)	
Non-Hispanic White	378 (37.1)	202 (33.6)	176 (42.2)	
Non-Hispanic Black	278 (27.3)	148 (24.6)	130 (31.2)	
Other Race	42 (4.1)	30 (5)	12 (2.9)	
HbA1c, %	7.5 ± 1.8	7.6 ± 1.9	7.2 ± 1.6	< 0.001
ALB, g/L	40.7 ± 3.7	41.0 ± 3.3	40.4 ± 4.1	0.023
BUN, mmol/L	5.6 ± 3.2	4.6 ± 1.5	7.2 ± 4.1	< 0.001
Scr, μmol/L	91.3 ± 55.1	74.3 ± 18.6	116.0 ± 76.9	< 0.001
eGFR, ml/min/1.73 m^2^	82.4 ± 39.6	94.3 ± 35.1	69.0 ± 40.1	< 0.001
ACR, g/mmol	1.6 (0.8, 5.1)	1.3 (0.7, 3.6)	2.5 (1.0, 10.1)	< 0.001
CHO, mmol/L	5.2 ± 1.3	5.3 ± 1.3	5.0 ± 1.3	0.002
TG, mmol/L	2.2 ± 2.1	2.2 ± 2.2	2.2 ± 1.9	0.671
UA, μmol/L	334.2 ± 94.3	306.9 ± 79.1	373.5 ± 100.4	< 0.001

Data are shown as the mean ± standard deviation, number (percentage) or median (interquartile range)

### Association of plasma homocysteine with CKD

**Fig. 2:**
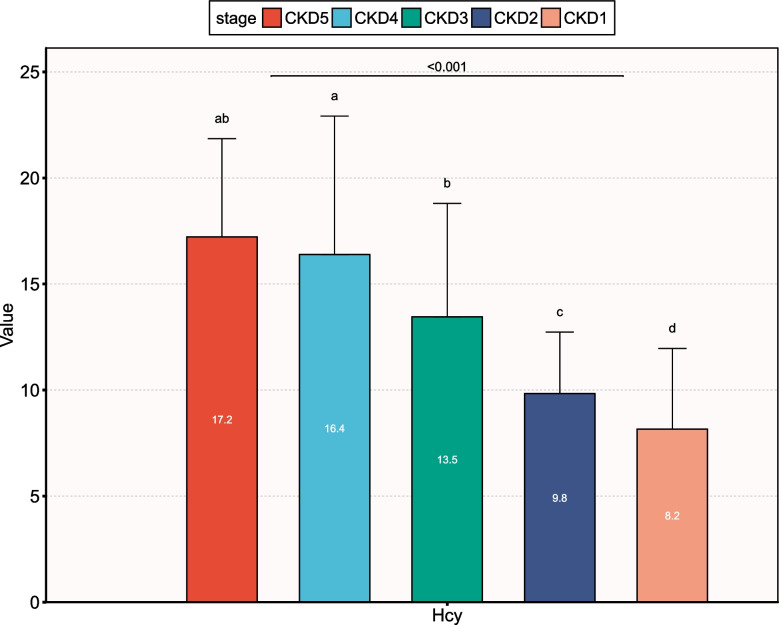
Plasma homocysteine levels at different stages of renal impairment in type 2 diabetes. CKD1 = eGFR ≥90 mL/min/1.73 m^2^; CKD 2 = eGFR Between 60 and 89 mL/min/1.73 m^2^; CKD 3 = eGFR between 30 and 59 mL/min/1.73 m^2^; CKD 4 = eGFR between 15 and 29 mL/min/1.73 m^2^; CKD 5 = eGFR<15 mL/min/1.73 m^2^. In CKD stage 1, the mean value of Hcy was 8.2 μmol/L. In CKD stage 1, the mean value of Hcy was 8.2 μmol/L. In CKD stage 2, the mean value of Hcy was 9.8 μmol/L. In CKD stage 3, the mean value of Hcy was 13.5 μmol/L. In CKD stage 4, the mean value of Hcy was 16.4 μmol/L. In CKD stage 5, the mean value of Hcy was 17.2 μmol/L

Table 3 showed the results of the univariate logistic regression analysis for the associations between Hcy and CKD. In the multivariate model that included age, race, HbA1c, ALB, CHO, TG and UA, the prevalence of CKD increased with increasing Hcy in all patients (OR, 1.17; 95% CI, 1.11–1.22; *P* <  0.001). For male and female patients, the ORs for CKD all tended to increase with increasing Hcy levels (male OR, 1.18; 95% CI, 1.1 ~ 1.27; *P* <  0.001; female OR, 1.15; 95% CI, 1.08 ~ 1.23; *P* <  0.001). Fig. 2 showed the homocysteine plasma levels at different stages of renal impairment in type 2 diabetes. Significant differences were observed among the five groups (*P* <  0.001), and the mean value of Hcy in the CKD5 stage was the highest.

**Table 3:** Logistic regression analysis of Hcy in relation to CKD risk

Variable	Male	*P* value	Female	*P* value	Total	
	OR (95% CI)		OR (95% CI)		OR (95% CI)	*P* value
Hcy						
Model 1	1.26(1.18 ~ 1.34)	< 0.001	1.2 (1.14 ~ 1.27)	< 0.001	1.23(1.18 ~ 1.29)	< 0.001
Mode 2	1.22(1.15 ~ 1.3)	< 0.001	1.17(1.11 ~ 1.24)	< 0.001	1.2 (1.15 ~ 1.25)	< 0.001
Model 3	1.18(1.1 ~ 1.27)	< 0.001	1.15(1.08 ~ 1.23)	< 0.001	1.17(1.11 ~ 1.22)	< 0.001

The data are presented as odds ratios (95% confidence intervals) and P values. Model 1: unadjusted; Model 2: adjusted for age and race; Model 3: adjusted for age, race, HbA1c, ALB, CHO, TG, and UA

## Discussion

In this cross-sectional study of 1018 patients with T2DM, we investigated the association between Hcy and the prevalence of CKD. We found that the prevalence of CKD in T2DM patients increased with higher Hcy value, independent of age, race, gender, and other clinical variables. The results also showed that gender, age, UA, TG, and HbA1c were associated with CKD. However, the associations among HbA1c, TG, and CKD were not statistically significant. Previously, diabetes, hypertension, hyperuricaemia, obesity, and hyperlipidaemia were identified as risk factors for CKD. This research have investigated the association between Hcy and CKD in US patients with T2DM.

In the last 10 years, Hcy has been considered as a marker of cardiovascular disease and a independent risk factor for many other diseases. HHcy is a trigger for many diseases, such as atherosclerosis, congestive heart failure, c, Alzheimer’s disease and hearing loss [14]. Several previous reports have assessed the association between Hcy and CKD. Li reported that Hcy is associated with tubular interstitial lesions in the early stages of IgA nephropathy [15]. Wang found that HHcy was more prevalent in patients with IgA nephropathy than in patients with other primary glomerular diseases, especially in the early stages of CKD, and may be a predictor of accelerated decline in renal function and future incidence of CKD [16]. Spence has shown that Hcy plays a significant role in the effect of renal dysfunction on atherosclerosis [17].

Diabetes mellitus is the most common cause of CKD. The hemodynamic changes and abnormal glucose metabolism caused by hyperglycemia are the basis of renal damage. However, The exact mechanisms underlying the association between HHcy and renal disease progression are not fully understood. Possible reasons are that HHcy inhibits vasodilation of renal arteries and promoting acceleration of progression of renal damage and glomerulosclerosis [18–20]. Increasing evidence indicated that there was a graded association between Hcy quartiles and eGFR decline. Compared with participants with the lowest quartile of Hcy levels, those with the highest quartile had significantly increased risk for rapid eGFR decline [21]. Figure. 2 indicated that renal function gradually deteriorated with increasing Hcy levels. An Israeli study found that subjects with homocysteine> 15 μmol/L were more likely to have an eGFR< 60 ml/min and proteinuria. At a GFR < 60 ml/min, homocysteine was progressively elevated in stages 3 and 4 of CKD [9, 22]. This is consistent with our findings. It was found that the longer the duration of T2DM, the higher the levels of Hcy in the plasma. A meta-analysis showed that Hcy levels were significantly higher in type 2 diabetic nephropathy (T2DN) patients with macro-albuminuria compared with T2DM patients without albuminuria and T2DN patients with micro-albuminuria, while Hcy status was significantly higher in T2DN patients with micro-albuminuria compared to T2DM patients without albuminuria [23].

This study had several limitations. Firstly, this was a retrospective cross-sectional study that did not confirm the causal relationship between Hcy and CKD in patients with T2DM. Secondly, the covariates in this study did not include medications that might affect the results of Hcy detection. Finally, there was a wide variation in the prevalence of MTHFR gene polymorphism across different populations around the world. MTHFR gene have been reported to be involved in the biological metabolism of folate, vitamin B12 and Hcy, maintaining DNA methylation patterns [24]. There was no genetic testing in this study, and the influence of MTHFR polymorphisms on the study results was unknown.

Nevertheless, the strength of this study was the relatively large sample size in patients with T2DM. This study provided useful and convincing epidemiological evidence for the association between Hcy and CKD in patients with T2DM. In conclusion, Hcy was found to be independently associated with CKD in patients with T2DM. In the future, a prospective cohort study is warranted to further investigate the impact of Hcy on CKD in patients with T2DM.

## Data Availability

Data can be downloaded from the ‘NHANES’database (https://www.cdc.gov/nchs/nhanes/index.htm).
